# Phytochemicals with Added Value from *Morella* and *Myrica* Species

**DOI:** 10.3390/molecules25246052

**Published:** 2020-12-21

**Authors:** Gonçalo P. Rosa, Bruno J. C. Silva, Ana M. L. Seca, Laila M. Moujir, Maria Carmo Barreto

**Affiliations:** 1 LAQV-REQUIMTE, Department of Chemistry, University of Aveiro, 3810-193 Aveiro, Portugal; goncalo.p.rosa@uac.pt (G.P.R.); ana.ml.seca@uac.pt (A.M.L.S.); 2 cE3c—Centre for Ecology, Evolution and Environmental Changes/Azorean Biodiversity Group, Rua Mãe de Deus, 9501-321 Ponta Delgada, Portugal; 3 Faculty of Sciences and Technology, University of Azores, Rua Mãe de Deus, 9501-321 Ponta Delgada, Portugal; brunosilva010575@hotmail.com; 4 Departamento de Bioquímica, Microbiología, Biología Celular y Genética, Facultad de Farmacia, Universidad de La Laguna, Avda Astrofísico Francisco Sánchez, s/n., 38200 La Laguna, Spain; lmoujir@ull.edu.es

**Keywords:** *Morella*, *Myrica*, myricanol, myricitrin, in vitro, in vivo

## Abstract

Terrestrial plants, due to their sessile nature, are highly exposed to environmental pressure and therefore need to produce very effective molecules that enable them to survive all the threats. *Myrica* and *Morella* (Myricaceae) are taxonomically close genera, which include species of trees or shrubs with edible fruits that exhibit relevant uses in traditional medicine. For instance, in Chinese or Japanese folk medicine, they are used to treat diarrhea, digestive problems, headache, burns, and skin diseases. A wide array of compounds isolated from different parts of *Myrica* and/or *Morella* species possess several biological activities, like anticancer, antidiabetic, anti-obesity, and cardio-/neuro-/hepatoprotective activities, both in vitro and in vivo, with myricanol, myricitrin, quercitrin, and betulin being the most promising. There are still many other compounds isolated from both genera whose biological activities have not been evaluated, which represents an excellent opportunity to discover new applications for those compounds and valorize *Morella*/*Myrica* species.

## 1. Introduction

Nature is an important source of new biologically active compounds and molecules with very diverse and unique biological properties and chemical structures, several of them being commercialized as medicines [1]. This diversity is the result of a long and selective evolutionary pressure [2], that led to an array of different biosynthetic pathways producing primary and, in particular, secondary metabolites with a large variety of basic skeletons and functional groups, which have been huge contributors to the improvement of human health [3].

That evolutionary pressure is more intense in plants and other sessile organisms because, since they are unable to move, they are more exposed to the action of herbivores, pathogens. and/or variable sunlight conditions, and therefore need to produce very effective molecules that enable them to survive all of these threats [4]. That fact, allied to the extraordinary biological and chemical diversity within plants, offers a particularly rich potential in biologically active compounds that can be used to provide lead compounds for the production of medications for treating various diseases from migraine to cancer. Twenty-five percent of all prescribed drugs are derived from plants, and of the 252 drugs considered as basic and essential by the World Health Organization, 11% are exclusively of plant origin and a significant number are drugs obtained from natural precursors by semi-synthesis. Thus, medicinal plants and their derived natural compounds are still an increasing topic of investigation and interest [5,6].

The members of the *Myrica* and *Morella* genera are woody shrubs or tree pioneers in nitrogen-poor soils such as sandy soil or gravelly sites, because they are actinorhizal plants able to fix nitrogen through nitrogen-fixing root nodules induced by soil actinomycetes of the genus *Frankia*, with which they establish a symbiotic relationship [7]. In addition to the economic interest of these species as sources of paper and rope from the bark, as fuel wood, for biomass production, and land reclamation, they are also appreciated because their fruits that can be eaten raw and are used in the production of jams, syrups, and juices [8], and their applications in traditional medicine are also noteworthy.

The *Myrica* genus comprised, before 2002, circa 97 species with a wide distribution in both temperate and sub-tropical regions [8,9]. Macdonald [10] presented various reasons for splitting this genus in two, *Myrica* and *Morella*, with his arguments only being accepted in 2002 [11]. A taxonomic key was published to allow the simple discrimination between species of the two genera [12]. The splitting of the *Myrica* genus led to many of the species previously belonging to the *Myrica* genus being reclassified and included in the *Morella* genus, which can cause problems when trying to correlate the discovery of secondary metabolites with the plant of origin. Many studies published before 2005 report the isolation of secondary metabolites from *Myrica* species which are now classified as *Morella* species, causing misleading reports on secondary metabolites found for the first time in the genus. Another issue is the fact that more recent publications use the previous scientific name, that consequently can lead to a compound not being properly detected in a literature survey. To prevent this from happening, the present work covers the secondary metabolites isolated from both genera and their respective biological activities. The botanical names of the species used in the present work are the ones currently accepted by the International Plant Names Index (IPNI) database, even if in the original publication the former name was used. In this case, the former name will appear in brackets.

## 2. Biological Activities Exhibited by Secondary Metabolites from *Morella* and *Myrica* Species

Several of the secondary metabolites isolated from *Morella* and *Myrica* species have been studied to evaluate their potential application in human health. Silva et al. [13] reviewed compounds isolated from *Morella* and *Myrica* species exhibiting antioxidant and anti-inflammatory activities, reporting the antioxidant potential of various compounds as well as pertinent structure/activity relationships that could direct future research in order to obtain new, more active, and safer molecules. Therefore, this section is focused on other biological activities exhibited by compounds isolated from species of the *Morella* and *Myrica* genera. There are two bioactive compounds, myricetin and arjunolic acid, isolated, among others, from *Myrica esculenta* Buch.-Ham. Ex D. Don [14], whose biological activities have already been revised by Gupta et al. [15] and Gosh et al. [16], ranging from antidiabetic to antibacterial, anticancer, or anti-inflammatory. For that reason, those compounds will not be addressed in this work.

### 2.1. In Vitro Activities

In vitro studies represent the first step in the evaluation of the pharmacological effects of compounds. They are a simpler and cheaper way to assess the bioactivities of the tested compounds and yield very important and relevant information to direct further investigations on the full pharmacological potential of a compound. The results of in vitro tests performed with compounds isolated from *Morella* and *Myrica* are summarized in Table 1, where the compounds are organized by family.

As seen in Table 1, bioactive compounds are found in every part of the plant, from roots to the leaves, and the same compound can be found in members of both genera, which shows a close chemotaxonomic relationship, resulting from the taxonomic reorganization of the previous genus *Myrica* [12].

A variety of compounds are present, including diarylheptanoids, dihydrochalcones, triterpenoids and flavonoids, but the majority of the bioactive compounds obtained from *Morella* and *Myrica* genera are cyclic diarylheptanoids, a class of compounds known by their wide array of biological activities [63]. From the analysis of Table 1 it is also clear that the compounds which have been more widely studied are myricanone (**1**), myricanol (**4**), quercitrin (**9**), myricitrin (**10**), and betulin (**16**), so details about the most interesting works and compounds from Table 1 will be discussed in the following paragraphs.

Myricanone (**1**) presents cytotoxic activity against A549 and HepG2 cell-lines with EC_50_ of 3.22 µg/mL [25], and 32.46 µM [27], respectively. Myricanone (**1**) increases the apoptotic rate of A549 tumor cells from 4.13% in untreated cells to 34.9% (after treatment) in a dose-dependent manner, and significantly inhibits colony formation in A-549, by inducing cell-cycle arrest in G1 phase [25]. Although the authors indicate that fluorouracil was used as positive control, and that myricanone (**1**) is more active than the positive control [25], the EC_50_ of fluorouracil is not presented, which hinders a more realistic assessment of the potency of myricanone effects against A549 cells.

Myricanol (**4**) showed cytotoxic activity against HL60 (leukemia), A549 (lung), and SK-BR-3 (breast) cell-lines, with IC_50_ similar or lower (Table 1) than the positive control cisplatin [18]. The author also demonstrated that induced apoptosis cell via mitochondrial (caspase 3, 8, and 9 were activated) and death receptor pathway in HL-60 cell, suggesting its potential anticancer activity. Similar results were obtained by Dai et al. [33] against A-549 cells.

Myricanol (**4**) also presents a very interesting activity by inhibiting muscle atrophy and dysfunction caused by dexamethasone in C2C12 myotubes [33]. As seen in Table 1, pretreatment with **4** induces a variety of processes that lead to or are a reflex of the direct activation of Sirtuin-1 [30], a protein that regulates skeletal muscle remodeling, which directly deacetylates and therefore activates PGC-1α to regulate mitochondrial biogenesis [64], induces autophagy to enhance degraded protein clearance and modulates FoxOs transcriptional activity to reduce atrogin-1 and MuRF1 expression, which are responsible for protein degradation, and consequently control loss of muscle mass and function [65]. Since there are only a few molecules approved for the treatment of muscle atrophy, and the majority come with very undesirable side effects like insulin resistance, sodium retention, or possibility of thromboembolisms [66], there is a need to find alternative molecules capable of treating this condition. The results shown by myricanol (**4**) turn it into a good candidate for that alternative.

Additionally, myricanol shows neuroprotective effects in N2a neuronal cells exposed to H_2_O_2_ [32]. Pretreatment with 0.84 mM of **4** increased 80% of cell viability, decreased 40% of intracellular reactive oxygen species (ROS) formation and reduced intracellular calcium concentrations, compared with the H_2_O_2_ group.

Quercitrin (**9**) is one of the most studied among the compounds from *Morella* and *Myrica* genera. Despite most of the studies being performed with quercitrin isolated from other sources, the fact that this compound has also been isolated in *Morella* and *Myrica* genera, namely *Morella adenophora* (Hance) J. Herb. [19], is in itself an added value to the biological importance of these genera. Compound **9** is a potent α-glucosidase inhibitor, an enzymatic target used to evaluate antidiabetic effect. It has an IC_50_ four times lower than acarbose, a commercial inhibitor, which indicates the great potential of quercitrin as a hypoglycemic agent for diabetes treatment [36]. Mercury and lead are considered as threats to human organisms, due to their low elimination rate and high accumulation, which have nefarious effects in several physiological functions, especially of liver and nervous system [67,68]. A study carried out by Aldana et al. [39] demonstrated the capacity of quercitrin (**9**) as cytoprotective agent for the HepG2 cell line against both metals. However, on PC12 cells, quercitrin was only able to protect against the effects of lead [39]. Further investigations are necessary to explain the cytoprotective mechanism of the compound.

Quercitrin (**9**) also presents a moderate antiviral activity by inhibition of neuraminidase (NA) [37], a viral enzyme common in human influenza viruses, which is essential for the virus release from infected cells to the neighboring cells of the respiratory tract [69]. It has also been reported that quercitrin (**9**) inhibits the activity of myeloperoxidase, with an IC_50_ of 2.0 µM [38], a result that hints at its possible role in reducing endothelial dysfunction and consequently that the protective effect of **9** should be evaluated in an atherosclerosis model.

A great number of the studies reviewed about the pharmacological activities of myricitrin (**10**) focused on its effects against cardiovascular problems [43,44,45]. Qin et al. [45] suggest that myricitrin treatment protected human umbilical vein endothelial cells (HUVECs) against the effects of oxidized low-density lipoprotein (ox-LDL), which results in reduced atherosclerotic plaque formation.

It was also found that 40 µM myricitrin protects H9c2 cardiomyocytes against hypoxia/reoxygenation injury, increasing cell-survival from 65.48% in untreated cells to 87.48% (Table 1) [43]. In addition, there was a reduction in the expression of pro-apoptotic factors, like caspase-3 or Bax, and an increase in the expression of anti-apoptotic factors, like Bcl-2. Likewise, the authors formulated that myricitrin (**10**) might exert its cardioprotective effects via stimulation of the expression of heat shock protein 90, whose activation promotes the anti-apoptotic PI3-K/Akt signaling pathway [43].

In a model of diabetic cardiomyopathy injury, Zhang et al. [44] found that pretreatment with 53.8 µM of myricitrin significantly decreased advanced glycation end products (AGEs)-induced inflammatory cytokine expression, limited an increase in ROS levels, and reduced cell apoptosis, fibrosis, and hypertrophy in H9c2 cells (Table 1). Overall, the protective effects of myricitrin could be attributed to its antioxidant and anti-inflammatory effects, as well as its ability to activate the Akt signaling pathway [43,44]. The anti-ROS activity of **10** is also responsible for the neuroprotective activities reported by Shen et al. [17] and Wang et al. [47] as observable by the effects described in Table 1.

Myricitrin (**10**) is also a potent α-glucosidase (IC_50_ = 1.1 µM) and β-amylase inhibitor (IC_50_ = 1.9 µM), being 20- and 10-fold more active than acarbose, respectively [48].

Myricanone (**1**), myricanol (**4**), and myricitrin (**10**) were found to inhibit melanogenesis in B16 mouse melanoma cells (Table 1) [26]. The melanin content found after exposure to the compounds was lower than the positive control, arbutin. However, the mechanism by which this occurred was different. For myricanone (**1**) and myricanol (**4**), it was due to their cytotoxic effects on the B16 cells, while **10** exerted its antimelanogenesis effect without killing the B16 cells, similar to the effect of arbutin [26].

Respiration uncoupling seems to be implicated in numerous physiological and pathological processes like autophagy, regulation of ROS production, protein secretion, capacity to carry out physical exercise, and adipose tissue biology [70]. As seen in Table 1, a series of dihydrochalcones, which have been isolated from various species of *Morella* and *Myrica*, possess the ability to uncouple the mitochondrial respiratory chain. Myrigalone A (**11**) and myrigalone G (**15**) were the best uncouplers, increasing the respiratory rate more than two-fold with respect to the positive control, 2,4-dinitrophenol (DNP) [53]. Although respiratory uncouplers may have some therapeutic interest, they must be evaluated for their safety, and there are no records of such study on the safety of myrigalones **11**–**15** as respiratory chain uncoupling agents.

Myrigalone B (**12**) and myrigalone G (**15**) inhibited α-glucosidase activity with IC_50_ of 19 and 7 µM, respectively, which is lower than the positive control, acarbose (43 µM). For β-amylase inhibition, **12** was again the most active with an IC_50_ of 8.3 µM against 33 µM obtained with compound **15** (Table 1) [48]. In this case myrigalone B (**12**) was still more active than acarbose, but not myrigalone G (**15**), since the IC_50_ of acarbose for β-amylase inhibition was 19 µM. Despite their potential as antidiabetic compounds, **12** and **15** are still less active inhibitors than myricitrin (**10**).

Another very interesting compound found in *Morella*/*Myrica* genera is betulin (**16**), which has presented a wide array of antitumor activities (Table 1). Lin et al. [57] reported the anticancer activity against two lines of osteosarcoma, MG-63 and HOS cell lines, with an IC_50_ of 14.54 and 11.70 µM, respectively. Unfortunately, no positive control was used to compare with the potency of botulin (**16**). The authors found that the effect of **16** in osteosarcoma cell lines is through the induction of apoptosis and autophagy to suppress cell viability, as well as by the inhibition of mTOR signaling. Betulin (**16**) is also very active against the NCi-H460 (IC_50_ = 2.8 µM) and HT29-MTX (IC_50_ = 1.6 µM) cell lines, being much more active than the positive control podophyllotoxin (22 and 24 µM against these two cell lines, respectively) [58]. Compound **16** was also found to be active against A549 and SK-Br-3 cell lines, with a more potent effect than cisplatin (Table 1), but is less potent against the HL60 cell-line [18].

Betulin (**16**) also exerts hepatoprotective activity against continuous ethanol exposure [59]. Ethanol-induced HSC-T6 cells treated with 25 µM of **16** suffered a decrease in the expression levels of collagen-I and α-SMA, which indicates that betulin (**16**) reduces ethanol-induced hepatic fibrosis. The expression levels of TLR4 were decreased after betulin pretreatment and there was an increased activation of STAT3. This modulation of TLR4 and STAT3 pathways is proposed as the mechanism through which **16** protects the hepatocytes against fibrosis.

A good number of the compounds from Table 1 were tested for their anti-tuberculosis activity against *Mycobacterium tuberculosis* H37Rv, (+)-Galeon (**8**) being the most active compound with a minimum inhibitory concentration (MIC) of 15 µg/mL. However, this value was still higher than the one obtained with the positive control, ethambutol, which has an MIC of 6.25 µg/mL [19]. The second-best compound, 5-deoxymyricanone (**2**), has an MIC of 25.8 µg/mL, which is more than four times the MIC of the positive control, so it cannot be considered very active.

*Morella*/*Myrica* compounds show remarkable pharmacological potential, with a variety of interesting in vitro activities. For further understanding of their full potential and clarification of mechanisms of action, their activities must be confirmed in vivo. The following section focuses on what is already known about the effects of compounds isolated from *Morella/Myrica* in living organisms.

### 2.2. In Vivo Tests

In vivo studies are necessary to understand the actual potential of compounds as future therapeutic agents. A few compounds isolated from *Morella*/*Myrica* genera reached the in vivo stage of evaluation of their pharmacological effects, which means that research teams recognize their potential and want to prove their full pharmacological value. The main results of those studies are summarized in Table 2, and the most relevant aspects are discussed below.

As expected, the three compounds which have been more extensively studied in vitro and which were reported to be bioactive were the ones selected by researchers to be evaluated concerning their in vivo activity (Table 2). These compounds are myricanol (**4**), myricitrin (**10**), and betulin (**16**), and many of the in vivo studies found in the present literature survey aimed to confirm or to clarify the mechanisms of action found on the in vitro studies.

A dosage of 5 mg/kg of myricanol (**4**) administered to C57BL/6 mice with dexamethasone-induced muscle wasting led to a reduction of muscle loss in both quadriceps and gastrocnemius muscle (Table 2). In addition, the mice from the treated group showed an improvement of grip strength of about 50 g and almost doubled the forced swim time when compared with the untreated group [30]. Muscle atrophy was inhibited, with muscle fiber diameter increasing almost by 25% when compared with the untreated group. As observed in the in vitro tests, myricanol (**4**) also prevents dexamethasone-induced muscle atrophy and weakness by activating SIRT1 to reduce muscle protein degradation, enhance autophagy, and promote mitochondrial biogenesis and function in mice [30].

In another study, myricanol (**4**) at a dosage of 25 mg/kg, when fed to C57BL/6J mice simultaneously with a high fat diet (HFD), was able to reduce body weight and body fat accumulation, when compared with mice fed only with the HFD [71]. Furthermore, compound **4** administration led to a 33% decrease in lipids serum levels and a 50% reduction of fasting insulin levels, which can be explained by the 2-fold increase observed in the insulin sensitivity on the treated group (Table 2). Other effects of **4** in the HFD-fed mice were the activation of a series of pathways, like adenosine monophosphate-activated protein kinase (AMPK), leading to the suppression of adipogenesis and induction of lipolysis and lipid combustion in adipocytes, or insulin receptor substrate 1 (IRS-1), which leads to mitochondrial biogenesis, increases mitochondrial oxidative metabolism, and benefits adenosine triphosphate (ATP) synthesis. Myricanol (**4**) also increases irisin serum levels, which induces mitochondrial oxidative metabolism, mitochondrial uncoupling, fatty acid oxidation in skeletal muscle [76], and increases energy expenditure in human adipocytes, resulting in reduced lipid accumulation [77]. Altogether, these data show this compound could be developed as a candidate for the treatment of insulin resistance and obesity. The effect of myricanol on lipid accumulation was confirmed by Shen et al. [31], but in a zebrafish model, with their findings showing the inhibition of lipid accumulation by suppressing adipogenic factors, including peroxisome proliferator activated receptor γ (PPARγ) and CCAAT/enhancer binding protein α (C/EBPα).

Myricanol (**4**) also decreased tumor growth in xenografted BALB/c nude mice at a dosage of 40 mg/kg, by activating the expression levels of pro-apoptotic factors within the tumor cells and downregulating the protein expression of Bcl-2, VEGF, HIF-1α, and survivin, thus increasing the apoptotic rate of tumor cells [72]. These effects led to a reduction in tumor volume of 39.4% after 14 days when compared with the untreated mice, showing the great potential of myricanol to become an anticancer drug.

In a murine model of atherosclerosis, a dosage of 50 mg/kg of myricitrin prevented the formation of Ox-LDL, which represented a reduction of 25% in serum levels of that atherosclerosis promoter [45]. A similar reduction was found in the mice treated with the positive control, probucol, but at a 40-fold higher dose than that of myricitrin, which means the latter is much more effective. By reducing the levels of Ox-LDL, myricitrin (**10**) led to a reduction of 20% in the expression of caspase-3 in the aortic arch, thus reducing endothelial cell apoptosis. This resulted in a complete inhibition of calcification in the aortic arch of treated mice, and a reduction on the atherosclerotic plaque area (Table 2) [45].

The in vivo investigation of the effects of myricitrin in a model of diabetic cardiomyopathy demonstrated that oral administration of a dosage of 300 mg/kg/day for 8 weeks remarkably decreased the expression of enzymes associated with cardiomyopathy, as well as the expression of inflammatory cytokines and apoptotic proteins [44]. This led to 20–25% improvement of diastolic dysfunction and attenuated histological abnormalities, by inhibiting the destruction of the collagen network, which reduces the fibrotic alterations of the heart. Mechanistically, the authors found that myricitrin (**10**) attenuated diabetes-induced Nrf2 inhibition via the regulation of Akt and extracellular-signal-related kinase (ERK) phosphorylation in the diabetic heart.

Another in vivo study showed that a dosage of 50 mg/kg of myricitrin (**10**) improved neuron injury and increased the expressions levels of PSD-95 protein and tyrosine hydroxylase TH protein in lipopolysaccharide (LPS)-stimulated BALB/c mice [74]. Loss of these proteins is found in Parkinson’s disease [78], so the fact that myricitrin increases their expression hints for a possible role in the prevention of this disease. In addition, myricitrin (**10**) decreased the production of pro-inflammatory factors including IL-1β, IL-6, and TNFα, decreased the level of chemokine MCP-1, and suppressed the expressions of COX-2 and iNOS (Table 2). Meanwhile, myricitrin suppressed HMGB1, TLR4, and MyD88 expression in the nigrostriatum of LPS-stimulated mice, and inhibited NF-κB and mitogen-activated protein kinase (MAPK) signaling pathways activated by LPS [74]. These effects show that myricitrin (**10**) is efficient in protecting the brain against inflammation and all the nefarious effects it causes.

Myricitrin (**10**) at a dosage of 100 mg/kg significantly ameliorated CCl_4_-induced hepatotoxicity in BALB/cN mice [73]. Treatment with this compound caused an increase in serum aspartate transaminase (AST) and alanine transaminase (ALT) levels and prevented histopathological changes in the liver (Table 2). Hepatic oxidative stress was reduced by myricitrin (**10**), as evidenced by the decrease in lipid peroxidation, with concomitant increase in glutathione (GSH) level and cytochrome P450 2E1 (CYP2E1) expression. Furthermore, cyclooxygenase-2 (COX-2) and tumor necrosis factor-alpha (TNF-a) overexpression in the liver was reduced, suggesting the suppression of inflammation [73]. Myricitrin (**10**) also improved the regeneration of hepatic tissue after CCl4-intoxication, as evidenced by increased proliferating cell nuclear antigen (PCNA) expression. The results presented by Domitrovic et al. [73] suggest that the anti-inflammatory and antioxidant effects of myricitrin (**10**) are responsible for its hepatoprotective activity. In fact, all the myricitrin (**10**) in vivo studies presented in Table 2 strongly indicate that **10** exerts its protective effects through the blockage of inflammation, oxidative stress, and apoptosis [44,45,73,74], which confirms myricitrin (**10**) as a potential candidate for the treatment and prevention of a wide array of disorders related to oxidative stress and inflammation.

Betulin (**16**) in vivo studies focused on its hepatoprotective effects against both chronic alcohol consumption [75] or acute ethanol induced fatty liver [47]. In the model of chronic consumption, administration of 20 mg/kg of betulin (**16**) to alcoholic C57BL/6 mice attenuated the increases in serum aminotransferase and triglyceride levels, while significantly inhibiting SREBP-1 expression and activating LKB1-AMPK phosphorylation [75]. Additionally, betulin (**16**) enhanced the sirtuin 1 (SIRT1) expression mediated by ethanol. Taken together, betulin alleviates alcoholic liver injury possibly through blocking the regulation of SREBP-1 on fatty acid synthesis and activating SIRT1-LKB1-AMPK signaling pathway.

In the acute ethanol-induced fatty liver model, administration of 50 mg/kg of betulin (**16**) to C57BL/6 mice induced a decrease in serum levels of hepatic enzymes and triglycerides [47]. The expression of SREBP-1, a transcription factor that promotes fatty acid synthesis [79] and whose activation by ethanol leads to fat accumulation on the liver, is reduced by betulin (**16**) to 75% of the expression levels presented by the untreated groups [47]. Betulin (**16**) administration also significantly decreased the expression levels of CYP2E1 and TLR4 and increased the activation of STAT3 (Table 2), thus impairing the ethanol induced pro-inflammatory response and, consequently reducing liver steatosis and fibrosis [47].

## 3. Other Phytochemicals Identified in *Morella* and *Myrica* Species

Many other secondary metabolites whose therapeutic application was not evaluated yet have been isolated in the last decades from *Morella* and *Myrica* species, constituting a pool of natural compounds structurally diverse with scientific interest, in which a research gap exists and which it is important to evaluate. These compounds are compiled in Table 3.

The pharmacological potential of the compounds presented in Table 3 has not been evaluated so far by the researchers who have studied *Morella* and *Myrica* species. It should be noted that the isolation and structural elucidation are the first steps to describing the metabolomic profile of a species or genus, helping to assess their potential as a source of commercially valuable phytochemicals, adding value to the species, as well as finding alternative sources of pharmacologically active metabolites.

The compounds listed in Table 3 belong to several families, like chalcones, dihydrochalcones, flavonoids, diterpenoids, triterpenoids, and diarylheptanoids, the latter two being the families with the highest number of compounds isolated. A similar tendency had already been observed in Table 1, where it was observed that diarylheptanoids were the most represented family, which indicates that *Morella* and *Myrica* genera are a good source of this family of compounds.

Since many of the unstudied compounds compiled in Table 3 share many structural features with some of the most active compounds that have already been assessed for their biological activities, there is plenty of potential for finding new added value compounds to increase the value of species from *Morella*/*Myrica* genera.

As an example, the only structural difference between myricanol (**4**) and myricananin B (**39**), two compounds isolated from the roots of *Morella nana* (A. Chev.) J. Herb. [21,28], is that the latter has an additional OH group at C-12. However, the biological activities of myricanol are widely studied, as reviewed in the present work, whereas the activities of myricananin B are not, which is unfortunate, since such structural similarity indicates a good pharmacological potential for myricananin B. A possible explanation is the fact that myricananin B (**39**) is obtained in low yields. In fact, starting with 20 kg of dried *Morella nana* (A. Chev.) J. Herb., Wang et al. [28] only obtained 5 mg of myricananin B, which according to the authors, represents a yield of 0.0000025%. With such a reduced amount of compound, it is understandable that myricananin B is so understudied, but the similarities with the bioactive myricanol point to a high pharmacological potential of compound **39**, so strategies to obtain higher amounts of this compound, like total or semi-synthesis should be employed.

Another example is the group of unstudied pentacyclic terpenes **57**–**70**. A compound from the same family, ursolic acid, was proven by in vivo and preclinical studies to possess multiple pharmacological activities, like anti-inflammatory, antitumor, cardioprotective, and antidiabetic, among others [85]. However, the poor water solubility and low intestinal absorption of ursolic acid, which leads to a rapid elimination by the gut wall/liver metabolism, resulting in low bioavailability [86,87], hinder the clinical applicability of ursolic acid. Some of the pentacyclic terpenes listed in Table 3, like arjunglucoside (**68**) and 3-*O*-(*E*)-caffeoyl-ursolic acid (**70**), present structural aspects that might overcome some of the limitations of the clinical use of ursolic acid, namely by being less lipophilic, which could increase their intestinal absorption and improve bioavailability.

These examples show that there are still many research opportunities in the study of *Morella*/*Myrica* and point out a great potential to increase the value of species belonging to these genera.

## 4. Conclusions

Compounds isolated from *Morella* and *Myrica* genera show great potential to become important pharmacological agents. Both in vitro and in vivo studies showed that compounds isolated from these genera, like myricanol (**4**), myricitrin (**10**), and betulin (**16**), are already seen as important candidates for the treatment of several diseases, namely due to their antitumor and cardio-/neuro-/hepatoprotective activities. Interestingly, in most cases their activity is related to their capacity to modulate oxidative stress and inflammation pathways, which increases their value even further, because of the myriad of pathologies related with those processes.

Although many of the studies presented their results with quality, there were some works where there was a lack of important information for a more correct interpretation of the validity of results. In some cases, authors present the IC_50_ without indicating the associated error, in others a positive control is not used, which hinders the perception of the true potency of the results. Future works should take these factors into consideration.

There are still many compounds isolated from *Morella* and *Myrica* genera whose biological activities have not been evaluated. However, since these compounds belong to chemical families with a high number of reported biological activities, a great potential for the chemical valorization of *Morella*/*Myrica* species can be foreseen, opening the door for future studies.

## Figures and Tables

**Table 1:** In vitro biological activities exhibited by compounds from *Morella* and *Myrica* species.

Compound	Origin	Biological Activities
Myricanone (**1**) 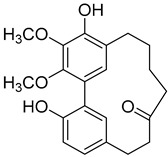	Methanol extract of *Myrica rubra* (Lour.) Siebold & Zucc. bark [17] Hexane extract of *Morella cerifera* (L.) Small (*Myrica cerífera)* bark [18] Methanol extract of *Morella adenophora* (Hance) J. Herb. roots [19] Chloroform and methanol extract of *Morella arborea* (Hutch.) Cheek twigs [20] 95% EtOH extract of *Morella nana* (A. Chev.) J. Herb. roots [21] Hexane extract of *Morella cerifera* (L.) Small (*Myrica cerífera)* twigs [22] Ethanol 95% extract of *Morella cerifera* (L.) Small (*Myrica cerífera)* bark [23] Methanol extract of *Myrica gale* L. (*Myrica gale* var *tormentosa* L.) branches [24]	Anti-tuberculosis [19] Minimum inhibitory concentration (MIC) = >150 µg/mL (Ethambutol MIC = 6.25 µg/mL). Cytotoxic activity [25] A549 cell line EC_50_ = 3.22 µg/mL (Fluorouracil EC_50_ = not shown). Increase in apoptotic rate to 34.9% at 5.0 µg/mL (4.13% on untreated cells). Antimelanogenesis activity [26] Melanin content in B16 mouse melanoma cells at 25 µg/mL: 23.5 ± 3.4% (arbutin 25 µg/mL: 77.4 ± 2.9%). Cell viability at 25 µg/mL: 17.7 ± 1.7% (arbutin 25 µg/mL: 102.0 ± 1.5%). Cytotoxic activity [27] HepG2 cell line: EC_50_ = 32.46 µg/mL. WRL-68 cell line: at 100 µg/mL cell viability was 89.27% (non-tumor cell line).
5-Deoxymyricanone (**2**) 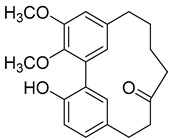	Methanol extract of *Morella adenophora* (Hance) J. Herb. roots [19]	Anti-tuberculosis [19] MIC = 25.8 µg/mL (Ethambutol MIC = 6.25 µg/mL).
12-Hydroxymyricanone (**3**) 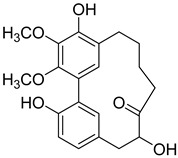	Methanol extract of *Morella adenophora* (Hance) J. Herb. roots [19] Ethanol 80% extracts of *Morella nana* (A. Chev.) J. Herb. roots [28] Methanol extract of *Myrica gale* L. (*Myrica gale* var *tormentosa* L.) branches [24]	Anti-tuberculosis [19] MIC = 35.8 µg/mL (Ethambutol MIC = 6.25 µg/mL).
Myricanol (**4**) 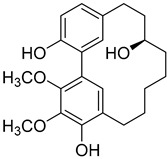	Methanol extract of *Myrica rubra* (Lour.) Siebold and Zucc. bark [17] Hexane extract of *Morella cerifera* (L.) Small (*Myrica cerífera)* bark [18] Methanol extract of *Morella adenophora* (Hance) J. Herb. roots [19] Methanol extract of *Myrica esculenta* Buch.-Ham. Ex D. Don leaves [14] Chloroform and methanol extract of *Morella arborea* (Hutch.) Cheek twigs [20] 95% EtOH extract of *Morella nana* (A. Chev.) J. Herb. roots [21] Dichloromethane: methanol (1:1) extract of *Morella arborea* (Hutch.) Cheek bark and stem [29] Ethanol 95% extract of *Morella cerifera* (L.) Small bark [23]	Inhibition of muscle atrophy and dysfunction [30] C2C12 myotubes treated with 10 µM myricanol and dexamethasone showed: ↑ myosin heavy chain expression (0.89 against 0.33 on cells treated only with dexamethasone), ↓ atrogin-1 expression (1.53 against 2.31 on cells treated only with dexamethasone), ↓ MuRF1 expression (0.99 against 1.55 on cells treated only with dexamethasone), ↑ ATP production (5.84 nM/mg protein against 3.83 nM/mg protein on cells treated only with dexamethasone), ↑ mitochondrial content (116.38% against 68.12% on cells treated only with dexamethasone), ↑ mitochondrial O_2_ consumption (223.77 pmol/min against 166.59 pmol/min on cells treated only with dexamethasone). Mitigation of lipid accumulation in 3TS-L1 adipocytes [31] Treatment with 5 µM myricanol increased AMPK activation in 50% when compared to the untreated cells. Decrease of 28% in lipid accumulation when compared to the untreated cells. Neuroprotective effects [32] 80% increase in N2a cells viability treated with 0.84 mM and 100 mM H_2_O_2_ (against cells treated only with H_2_O_2_). 40% reduction of intracellular ROS in N2a cells treated with 0.84 mM and 100 mM H_2_O_2_ when compared with the cells treated only with H_2_O_2_. Intracellular calcium concentration of 777.81 nM in N2a cells treated with 0.84 mM and 100 mM H_2_O_2_ (3045.51 nM in cells treated only with H_2_O_2_). Antitumor activity [18] HL60 cell-line: IC_50_ = 5.3 ± 0.7 µM (Cisplatin EC_50_ = 4.2 ± 1.1 µM). A549 cell-line: IC_50_ = 16.5 ± 0.8 µM (Cisplatin EC_50_ = 18.4 ± 1.9 µM). SK-BR-3 cell-line: EC_50_ = 14.8 ± 5.5 µM (Cisplatin EC_50_ = 18.8 ± 0.6 µM). Anti tuberculosis [19] MIC = 30 µg/mL (Ethambutol MIC = 6.25 µg/mL). Cytotoxic activity [33] A549 cell line EC_50_ = 4.85 µg/mL (Fluorouracil EC_50_ = not shown). Antimelanogenesis activity [26] Melanin content in B16 mouse melanoma cells at 25 µg/mL: 3.8 ± 0.4% (arbutin 25 µg/mL: 77.4 ± 2.9%). Cell viability at 25 µg/mL: 8.8 ± 0.2% (arbutin 25 µg/mL: 102.0 ± 1.5%).
Myricanol 11-sulphate (**5**) 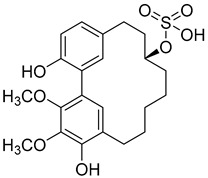	Methanol extract of *Myrica rubra* (Lour.) Siebold and Zucc. bark [17]	Protection against glutamate-induced damage [17] PC12 cell line: 5 µM of myricanol 11-sulphate maintained 72.09 ± 2.09% of cell viability after 24 h exposure to glutamate. (Cells treated only with glutamate showed viability of 50%).
Porson (**6**) 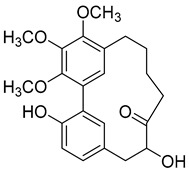	Methanol extract of *Morella adenophora* (Hance) J. Herb. roots [19] 95% EtOH extract of *Morella nana* (A. Chev.) J. Herb. roots [21] Methanol extract of *Myrica gale* L. (*Myrica gale* var *tormentosa* L.) branches [24] Ethyl acetate extract of *Myrica gale* L. stems [34]	Anti-tuberculosis [19] MIC = 40 µg/mL (Ethambutol MIC = 6.25 µg/mL).
Myricananin C (**7**) 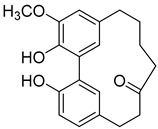	Methanol extract of *Morella adenophora* (Hance) J. Herb. roots [19] Ethanol 80% extract of *Morella nana* (A. Chev.) J. Herb. roots [28]	Anti-tuberculosis [19] MIC = 55.5 µg/mL (Ethambutol MIC = 6.25 µg/mL).
(+)-Galeon (**8**) 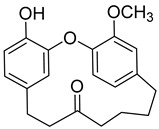	Methanol extract of *Morella adenophora* (Hance) J. Herb. roots [19] Methanol extract of *Myrica gale* L. (*Myrica gale* var *tormentosa* L.) branches [35] Ethyl acetate extract of *Myrica gale* L. stems [34]	Anti-tuberculosis [19] MIC = 15.0 µg/mL (Ethambutol MIC = 6.25 µg/mL).
Quercitrin (**9**) 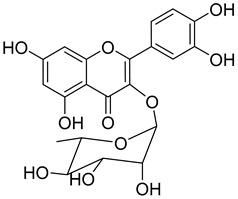	Methanol extract of *Myrica rubra* (Lour.) Siebold and Zucc. bark [17] Methanol extract of *Morella adenophora* (Hance) J. Herb. roots [19]	α-Glucosidase Inhibition [36] IC_50_ = 0.231 ± 0.033 mg/mL (Acarbose IC_50_ = 1.457 ± 0.144 mg/mL). Antiviral Activity [37] Neuraminidase inhibition: IC_50_ = 311.76 μM (Oseltamivir acid IC_50_ = 280 μM). Myeloperoxidase inhibition [38] IC_50_ = 2.0 ± 0.2 μM Prevention of metal toxicity effects [39] Almost 100% HepG2 cell line viability when treated with 1 μM + 15 μM MeHg (50% viability when only treated with 15 μM MeHg). Almost 100% HepG2 cell line viability when treated with 1 μM + 40 μM Pb(NO_3_)_2_ (50% viability when only treated with 40 μM Pb(NO_3_)_2_). About 50% PC12 cell line viability when treated with 1 μM + 10 μM MeHg (65% viability when only treated with 10 μM MeHg). Almost 90% PC12 cell line viability when treated with 1 μM + 350 μM Pb(NO_3_)_2_ (60% viability when only treated with 350 μM Pb(NO_3_)_2_). Anti-tuberculosis [19] MIC = >150 µg/mL (Ethambutol MIC = 6.25 µg/mL).
Myricitrin (**10**) 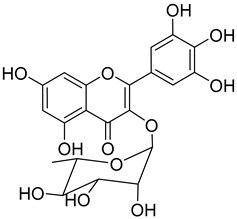	Methanol extract of *Myrica rubra* (Lour.) Siebold and Zucc. bark [17] Methanol extract of *Morella adenophora* (Hance) J. Herb. roots [19] Toluene extract of the root-bark *Morella cerifera* (L.) Small (*Myrica cerifera)* [40] Methanol extract of *Myrica esculenta* Buch.-Ham. Ex D. Don leaves [14] 80% Acetone extract of *Myrica rubra* (Lour.) Siebold and Zucc. leaves [41] Benzene extract (after petroleum ether) of *Morella cerifera* (L.) Small (*Myrica cerifera)* root bark [42]	Protection against glutamate-induced damage [17] PC12 cell line: 10 µM of myricitrin maintained 73.15 ± 3.23% of cell viability after 24 h exposure to glutamate. (Cells treated only with glutamate showed only viability of 50%). Protection against hypoxia/reoxygenation (R/H) injury in cardiomyocyte [43] H9c2 cells pretreated for 12 h with 40 µM had a survival rate of 87.48 ± 7.68% (survival rate of untreated cells = 65.48 ± 6.42%). 50% decrease of LDH levels secreted to culture medium in the cells pretreated for 12 h with 40 µM when compared with untreated cells. Pretreatment with 40 µM inhibited intracellular ROS generation in 50% when compared to untreated cells. Reduction of 50% in the apoptotic rate on cells treated with 40 µM when compared with the untreated cells. Pretreatment with 40 µM decreased the caspase-3 activity in 25% when compared with the untreated cells. Pretreatment with 40 µM increased the Bcl-2/Bax expression ratio to 1.28 ± 0.31 (0.46 ± 0.12 in untreated cells. Protection against advanced glycation end products (AGEs) injury in cardiomyocyte [44] Pretreatment with 25 µg/mL led to H9c2 cells viability of 78.94 ± 4.52% (60.34 ± 6.52% when treated only with 400 μg/mL AGEs). Pretreatment with 25 µg/mL µM decreased phospho-IKK-β expression in about 40%, when compared with the untreated cells. Pretreatment with 25 µg/mL decreased TNF-α expression in about 50%, when compared with the untreated cells. Pretreatment with 25 µg/mL decreased intracellular ROS generation in about 50%, when compared with the untreated cells. Pretreatment with 25 µg/mL markedly attenuated the inhibition of NQO-1, γ -GCS, and HO-1 expression induced by AGEs. Pretreatment with 25 µg/mL decreased caspase-3 and caspase-9 activity in 3 and 2-fold, respectively, when compared with the untreated cells. Pretreatment with 25 µg/mL decreased apoptotic rate in about 7%, when compared with the untreated cells. Pretreatment with 25 µg/mL led to a 2-fold decrease in the expression of Bax compared with the untreated cells. Pretreatment with 25 µg/mL led to a 2-fold increase in the expression of Bcl-2 compared with the untreated cells. Pretreatment with 25 µg/mL led to a 33% decrease in the expression of collagen-1 compared with the untreated cells. Prevention of atherosclerosis [45] Pretreatment with 40 µM in oxidized low-density lipoprotein (ox-LDL) exposed human umbilical vein endothelial cells (HUVECs): Increased cell viability (70.75 ± 8.44% against 50.25 ± 7.95% in the untreated cells); Reduction of apoptotic cells (15.58 ± 4.65% against 23.89 ± 3.65% in the untreated cells); 3-fold reduction of intracellular ROS levels when compared with untreated cells; ~33% reduction on caspase-3 activity when compared with untreated cells. Anti-tuberculosis [19] MIC = >150 µg/mL (Ethambutol MIC = 6.25 µg/mL). Protection against acrylamide induced oxidative stress [46] Acrylamide induced Caco-2 cells treated with 40 µg/mL presented a viability of 80% (untreated cells had a viability of 50%). Neuroprotective activity [47] Pretreatment with 10µM prevents PC12 cell death induced by 6-hydroxydopamine (6-OHDA) (untreated cells presented 25% of cell mortality). Pretreatment with 10µM decreased cytochrome C release by almost 60% when compared with the untreated group. Pretreatment with 10µM decreased caspase-3 activity release by almost 50% when compared with the untreated group. Pretreatment with 10µM decreased the apoptotic rate activity by almost 50% when compared with the untreated group. Antidiabetic activity [48] α-glucosidase inhibition: IC_50_ = 1.1 ± 0.06 µM (acarbose: IC_50_ = 43 ± 1.6 µM). β-amylase inhibition: IC_50_ = 1.9 ± 0.02 µM. (acarbose: IC_50_ = 19 ± 1.6 µM). Antimelanogenesis activity [26] Melanin content in B16 mouse melanoma cells at 25 µg/mL: 69.9 ± 2.3% (arbutin 25 µg/mL: 77.4 ± 2.9%). Cell viability at 25 µg/mL: 102.9 ± 3.3% (arbutin 25 µg/mL: 102.0 ± 1.5%).
Myrigalone A (**11**) 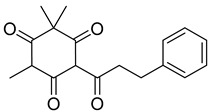	50% acetone extract of *Myrica gale* L. seeds [49] Methanol extract of *Myrica gale* L. leaves and fruits [34], fruits [50], seeds [51] Diethyl ether extract of the fruit exudate of *Myrica gale* L. [52]	Uncoupling activity [53] Increases mitochondrial respiration rate by 87 ± 8 natoms O/min/mg (DNP 36 ± 3 natoms O/min/mg) at 45 µM.
Myrigalone B (**12**) 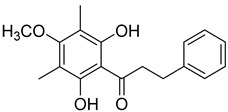	50% Acetone extract of *Myrica gale* L. seeds [49] Methanol extract of *Myrica gale* L. seeds [54] Dichloromethane extract of *Morella serrata* (Lam.) Killick leaves [55] Diethyl ether extract of the fruit exudate of *Myrica gale* L. [52]	Antidiabetic activity [48] α-glucosidase inhibition: IC_50_ = 19 ± 1.0 µM (acarbose: IC_50_ = 43 ± 1.6 µM). β-amylase inhibition: IC_50_ = 8.3 ± 1.3 µM (acarbose: IC_50_ = 19 ± 1.6 µM). Uncoupling activity [53] Increases mitochondrial respiration rate by 40 ± 10 natoms O/min/mg (DNP 36 ± 3 natoms O/min/mg) at 45µM.
Myrigalone D (**13**) 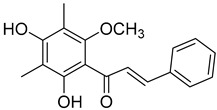	50% acetone extract of *Myrica gale* L. seeds [49] Methanol extract of *Myrica gale* L. seeds [54] and leaves [34] From the *Myrica gale* L. fruits [53]	Uncoupling activity [53] Increases mitochondrial respiration rate by 14 ± 2 natoms O/min/mg (DNP 36 ± 3 natoms O/min/mg) at 45 µM.
Myrigalone H (**14**) 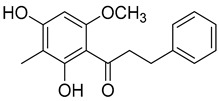	Diethyl ether extract of the fruit exudate of *Myrica gale* L. [52] *Myrica gale* L. fruit exudate [56]	Uncoupling activity [53] Increases mitochondrial respiration rate by 17 ± 3 natoms O/min/mg (DNP 36 ± 3 natoms O/min/mg) at 45 µM.
Myrigalone G (**15**) 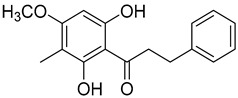	50% acetone extract of *Myrica gale* L. seeds [49] Diethyl ether extract of the fruit exudate of *Myrica gale* L. [52]	Antidiabetic activity [48] α-glucosidase inhibition: IC_50_ = 7 ± 1.4 µM (acarbose: IC_50_ = 43 ± 1.6 µM). β-amylase inhibition: IC_50_ = 33 ± 6.6 µM (acarbose: IC_50_ = 19 ± 1.6 µM). Uncoupling activity [53] Increases mitochondrial respiration rate by 71 ± 5 natoms O/min/mg (DNP 36 ± 3 natoms O/min/mg) at 45 µM.
Betulin (**16**) 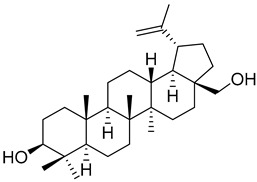	Hexane extract of *Morella cerifera* (L.) Small (*Myrica cerifera)* bark [18]	Anti-osteosarcoma activity [57] MG-63 cell line: EC_50_ = 14.54 µM. HOS cell line: EC_50_ = 11.70 µM. Antitumor activity [58] NCI-H460 cell line: EC_50_ = 2.8 ± 0.4 µM (Podophyllotoxin: EC_50_ = 22 ± 6 µM). HT29-MTX cell line: EC_50_ = 1.6 ± 0.4 µM (Podophyllotoxin: EC_50_ = 24 ± 3 µM). Antitumor activity [18] HL60 cell-line: EC_50_ = 15.2 ± 2.3 µM (Cisplatin EC_50_ = 4.2 ± 1.1 µM). A549 cell-line: EC_50_ = 5.2 ± 3.0 µM (Cisplatin EC_50_ = 18.4 ± 1.9 µM). SK-BR-3 cell-line: EC_50_ = 3.1 ± 0.6 µM (Cisplatin EC_50_ = 18.8 ± 0.6 µM). Hepatoprotective activity [59] Pretreatment with 25 µM in ethanol-induced HSC-T6 cells: Decrease in the expression levels of collagen-I (35%), α-SMA (25%) and TLR4 (60%), when compared with untreated cells. ~30% increase in phosphorylation of STAT3, when compared with the untreated group.
Myriceric acid A (**17**) 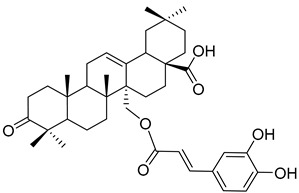	Methanol extract of *Morella cerifera* (L.) Small (*Myrica cerifera)* twigs [60] and branches [61]	Anti-hypertension activity [60] Endothelin 1 receptor antagonist: IC_50_ = 11 ± 2 nM.
3β-*trans*-*p*-Coumaroyloxy-2α,23-dihydroxyolean-12-en-28-oic acid (**18**) 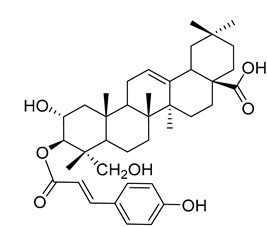	Methanol extract of *Morella adenophora* (Hance) J. Herb. roots [19]	Anti-tuberculosis [19] MIC = 45 µg/mL (Ethambutol MIC = 6.25 µg/mL).
(*R*)-4-(5-Hydroxy-7-(4-hydroxyphenyl)heptyl)-2-methoxyphenol (**19**) 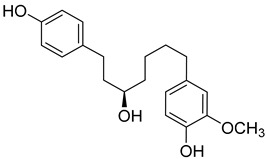	Methanol extract of *Morella adenophora* (Hance) J. Herb. roots [19]	Anti-tuberculosis [19] MIC = 52 µg/mL (Ethambutol MIC = 6.25 µg/mL).
Corchoionoside C (**20**) 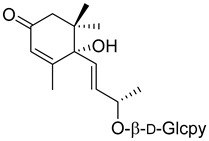	Methanol extract of *Myrica esculenta* Buch.-Ham. Ex D. Don leaves [14]	Angiotensin I-converting enzyme [14] 29.97 ± 4.77% ACE 1 inhibition at 100 µM (Captopril 88.64 ± 2.57 at 4.6 µM).
(6*S*,9*R*)-Roseoside (**21**) 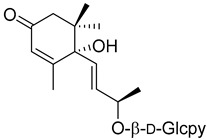	Methanol extract of *Myrica esculenta* Buch.-Ham. Ex D. Don leaves [14]	Antimelanogenesis activity [62] Inhibition of melanogenesis in B16 mouse melanoma cells at 100 µM: 62.7 ± 3.1% (arbutin 100 µM: 70.3 ± 5.5%). Cell viability at 100 µM: 95.0 ± 2.2% (arbutin 100 µM: 87.5 ± 2.8%). Angiotensin I-converting enzyme [14] 25.63 ± 1.35% ACE 1 inhibition at 100 µM (Captopril 88.64 ± 2.57 at 4.6 µM).
Myricadenin A (**22**) 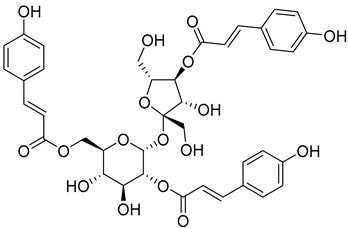	Methanol extract of *Morella adenophora* (Hance) J. Herb. roots [19]	Anti-tuberculosis [19] MIC = 80.0 µg/mL (Ethambutol MIC = 6.25 µg/mL).

d-Glcpy = d-glucopyranoside.

**Table 2:** In vivo biological activities exhibited by compounds from *Morella* and *Myrica* species.

Compound	Model	Dose	Activity
Myricanol (**4**)	C57BL/6 mice	5 mg/kg	Protection against muscle atrophy [30] Reduction of quadriceps muscle mass loss (1.36 ± 0.02% b/w against 1.18 ± 0.06% b/w on the untreated group). Reduction of gastrocnemius muscle mass loss (0.87 ± 0.08% b/w against 0.78 ± 0.05% b/w on the untreated group). Improvement of grip strength (120.58 ± 7.93 g against 70.90 ± 04.59 g on the untreated group). Increase in forced swim time (83.75 ± 15.19 s against 48.80 ± 11.43 s on the untreated group). Inhibition of muscle atrophy (~25% increase in muscle fiber diameter when compared with untreated group).
	C57BL/6J	25 mg/kg	Anti-obesity and diabetic activity [71] Reduction of body weight and body fat gain under high fat diet when compared with untreated group. Decrease in serum total cholesterol, triglycerides, LDL-cholesterol, and LDL/HDL ratio (~33%). 50% reduction of fasting insulin levels when compared with untreated group. ~2-fold increase in insulin sensitivity when compared with untreated group. Increased levels of phosphorylation of IRS-1 (2.5-fold), AKT (3-fold), and GSK-3β (1.5-fold) when compared with untreated group. 35% decrease in adipocyte diameter when compared with untreated group. ~33% increase in irisin serum levels when compared with untreated group.
	Zebrafish	1 µM	Anti-obesity activity [31] 66% decrease in lipid accumulation under high-fat diet, when compared with the untreated group (positive control AICAR (5 µM): 75% decrease). ~70–80% reduction of PPAR-γ, C/EBPα, SREB-1 and aP2 expression when compared with the untreated group (similar values obtained with the positive control).
	BALB/c nude mice	40 mg/kg	Antitumor activity [72] 39.4% reduction on A549 xenograft tumor volume after 14 days, when compared with untreated group. ~33% increase in the expression levels of Bax when compared with untreated group. Decrease in the expression levels of Bcl-2 (25%), vascular endothelial growth factor (VEGF) (20%), and Survivin (33%) when compared with untreated group. 20% increase on the number of apoptotic tumor cells when compared with untreated group.
Myricitrin (**10**)	ApoE -/- mice	50 mg/kg	Prevention of atherosclerosis [45] ~25% reduction of serum levels of Ox-LDL (similar reduction in the positive control group treated with 2 g/kg of probucol). 22% reduction on aortic wall thickness when compared to the untreated group. Complete inhibition of calcification on aortic arch. (Calcification observed in the untreated group). 26% reduction on atherosclerotic plaque area when compared with the untreated group. ~20% reduction of caspase-3 expression in aortic arch.
	BALB/cN mice	100 mg/kg	Hepatoprotective activity [73] Protection against CCl_4_ intoxication. Reduction in body weight change (−12.9 ± 0.8% against 19.6 ± 2.3% on the untreated group. Reduction of ALT serum levels (481 ± 53 U/L against 2126 ± 268 U/L on the untreated group). Reduction of AST serum levels (592 ± 74 U/L against 2538 ± 322 U/L on the untreated group). Reduction of liver lipids peroxidation. Increase on glutathione levels (46.1 ± 3.4 µmol/g protein against 28.6 ± 2.8 µmol/g protein on the untreated group). ~25% decrease in necrotic areas on the liver when compared with untreated group. 5-fold increase on CYP2E1 expression when compared with the untreated group.
	BALB/c mice	300 mg/kg	Protection against diabetic cardiomyopathy [44] ~20–25% improvement in cardiac function of diabetic mice when compared with untreated group. Decrease on abnormalities in the arrangement of cardiac fibers and morphology of cardiomyocytes when compared with untreated group. Inhibition of collagen network destruction and reduction of fibrotic alterations of the hearth. 2-fold reduction in the expression of TGF-β1 when compared with the untreated group. 8-fold reduction in the expression levels of collagen-1. Reduction on serum levels of IL-6 (35.72 pg/mL against 112.41 pg/mL in the untreated group). Reduction on serum levels of TNF-α (24.83 pg/mL against 56.21 pg/mL in the untreated group). Decrease of Bax/Bcl-2 ratio (35-fold), caspase-3 (5-fold), and caspase-9 (4-fold) expression levels, when compared with the untreated group.
	BALB/c mice	50 mg/kg	Neuroprotective activity [74] In LPS-stimulated mice: ~2-fold increase on the expression levels of PSD-95 and TH, when compared with untreated group. Reduction on expression levels of IL-1β (3-fold), IL-6 (2-fold), TNF-α (2-fold), and MCP-1 (2-fold), when compared with the untreated group. Reduction on the expression levels of COX-2 (3-fold) and iNOS (2-fold), when compared with the untreated group. Suppression of LPS-stimulated p38 (4-fold), ERK (4-fold), and JNK (2-fold) activation.
Betulin (**16**)	C57BL/6 mice	20 mg/kg	Hepatoprotective activity [75] Decrease of liver/body weight ratio in alcoholic mice, when compared with untreated group. Decrease in serum levels of ALT (66%), AST (33%), and TG (50%), when compared with the untreated group. Decrease on expression levels of collagen-I (20%), SREBP-1 (25%), and α-SMA (50%), when compared with untreated group. Increased phosphorylation of LKB1 (15%) and AMPK (25%) when compared with untreated group. 7-fold increase in SIRT-1 expression when compared with untreated group.
	C57BL/6 mice	50 mg/kg	Hepatoprotective activity [47] Decrease in serum levels of ALT (50%), AST (50%), and TG (33%) in ethanol induced fatty-liver mice, when compared with the untreated group. Decrease in expression levels of SREBP-1 (75%), CYP2E1 (60%), and TLR4 (60%), when compared with the untreated group. ~30% increase in phosphorylation of STAT3, when compared with the untreated group.

**Table 3:** Other secondary metabolites isolated from *Morella*/*Myrica* genera.

Name	Structure	Extract, Species, and Part of Plant
2′,4′-Dihydroxy-6′-methoxy-3′,5′-dimethylchalcone (**23**)	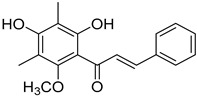	Dichloromethane extract of *Morella serrata* (Lam.) Killick leaves [55]
Aurentiacin A (**24**)	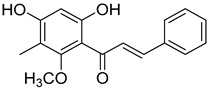	Dichloromethane extract of *Morella serrata* (Lam.) Killick leaves [55]
2′,6′-Dihydroxy-4′-methoxy-3′-methyl-dihydrochalcone (**25**)	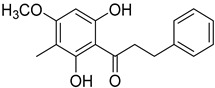	Dichloromethane extract of *Morella serrata* (Lam.) Killick leaves [55]
Myrigalone E (**26**)	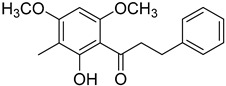	Methanol extract of *Myrica gale* L. seeds [54] From the *Myrica gale* L. fruits [53] Dichloromethane extract of *Morella serrata* (Lam.) Killick leaves [55]
Myrigalone P (**27**)	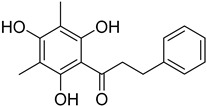	Methanol extract of *Myrica gale* L. seeds [54]
Uvangoletin (**28**)	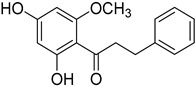	Methanol extract of *Myrica gale* L. seeds [54]
Angoletin (**29**)	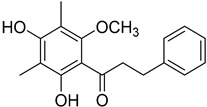	Fruit exudate from the *Myrica gale* L. fruits [53,56]
Demethoxymatteucinol (**30**)	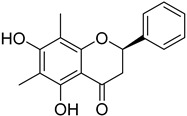	Dichloromethane extract of *Morella serrata* (Lam.) Killick leaves [55] Methanol extract of *Myrica gale* L. seeds [54]
Cryptostrobin (**31**)	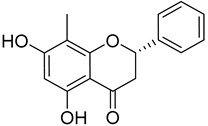	Dichloromethane extract of *Morella serrata* (Lam.) Killick leaves [55]
Demethoximatteucinol-7-methoxy (**32**)	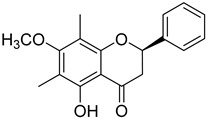	Methanol extract of *Myrica gale* L. seeds [54]
Myricetin 3-*O*-6″-galloyl-β-D-galactopyranoside (**33**)	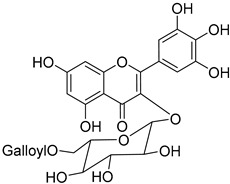	Methanol extract of *Myrica gale* L. (*Myrica gale* var *tormentosa* L.) branches [24]
Gallocatechin-4-α-8-epicatechin (**34**)	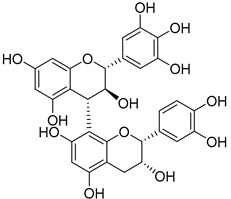	Aerial parts of *Myrica gale* L. [80]
Gallocatechin-4-α-8-epigallocatechin (**35**)	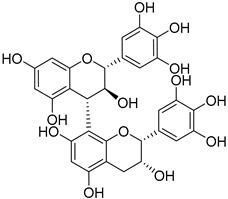	Aerial parts of *Myrica gale* L. [80]
Adenodimerin B (**36**)	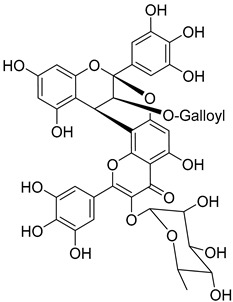	Methanol extract of *Morella adenophora* (Hance) J. Herb. roots [19]
Adenodimerin C (**37**)	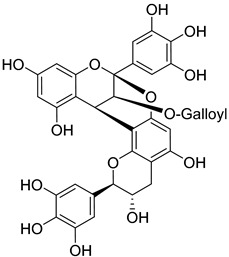	Methanol extract of *Morella adenophora* (Hance) J. Herb. roots [19]
Gallocatechin-(4-α-8)-gallocatechin-(4-α-8)-gallocatechin (**38**)	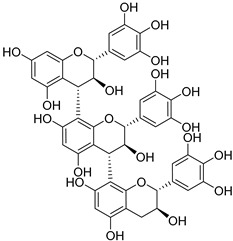	Aerial parts of *Myrica gale* L. [80]
Myricananin B (**39**)	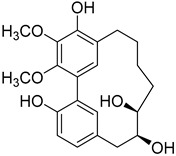	Ethanol 80% extracts of *Morella nana* (A. Chev.) J. Herb. roots [28]
Myricananin E (**40**)	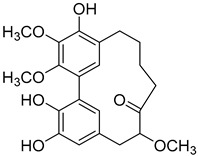	Ethanol 80% extracts of *Morella nana* (A. Chev.) J. Herb. roots [28]
Myricananin F (**41**) *	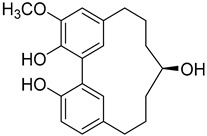	80% ethanol extracts of *Morella nana* (A. Chev.) J. Herb. roots [81]
Myricananin G (**42**)	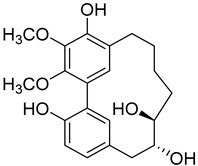	80% ethanol extracts of the of *Morella nana* (A. Chev.) J. Herb. roots [81]
Myricananin H (**43**)	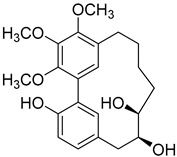	80% ethanol extracts of *Morella nana* (A. Chev.) J. Herb. roots [81]
Myricatomentogenin (**44**)	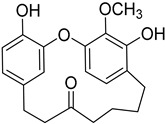	Ethanol 80% extracts of *Morella nana* (A. Chev.) J. Herb. roots [81]
Myricanone 5-*O*-β-D-glucopyranoside (**45**)	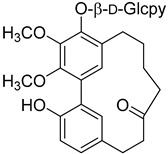	Methanol extract of *Morella adenophora* (Hance) J. Herb. roots [19] 80% ethanol extract of *Morella nana* (A. Chev.) J. Herb. roots [81]
Acerogenin 2-methyl ether (**46**)	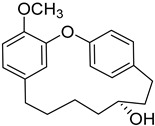	80% ethanol extract of *Morella nana* A. Chev.) J. Herb. roots [81]
Myricananone (**47**)	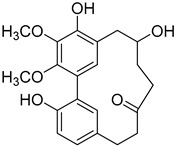	95% EtOH extract of *Morella nana* A. Chev.) J. Herb. roots [21]
Myricananadiol (**48**)	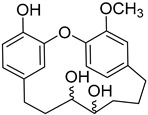	95% EtOH extract of *Morella nana* A. Chev.) J. Herb. roots [21]
Myricanol 11-*O*-β-D-xylopyranosyl (**49**)	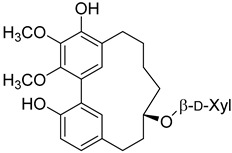	Methanol extract of *Morella adenophora* (Hance) J. Herb. roots [19] Dichloromethane: methanol (1:1) extract in of *Morella arborea* (Hutch.) Cheek bark and stem [29]
Myricatomentoside I (**50**)	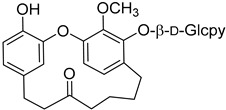	Methanol extract of *Myrica gale* L. (*Myrica gale* var *tormentosa* L.) branches [35]
Myricatomentoside II (**51**)	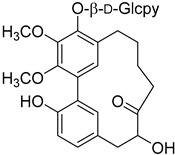	Methanol extract of *Myrica gale* L. (*Myrica gale* var *tormentosa* L.) branches [35]
12-Dehydroporson (**52**)	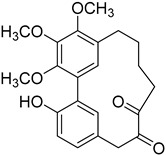	Methanol extract of *Myrica gale* L. (*Myrica gale* var *tormentosa* L.) branches [24]
Myricarborin (**53**)	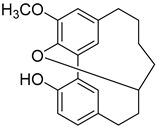	Dichloromethane: methanol (1:1) extract of *Morella arborea* (Hutch.) Cheek bark and stem [29]
Myricanene A 5-*O*-α-L-arabinofuranosyl (1→6)-β-D-glucopyranoside (**54**)	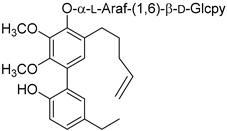	Methanol extract of *Morella adenophora* (Hance) J. Herb. roots [19]
Myresculoside (**55**)	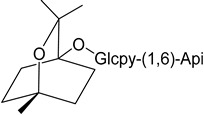	Methanol extract of *Myrica esculenta* Buch.-Ham. ex D. Don leaves [14]
(1*S*,2*S*,4*R*)-2-Hydroxy-1,8-cineole β-D-glucopyranoside (**56**)	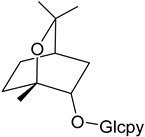	Methanol extract of *Myrica esculenta* Buch.-Ham. ex D. Don leaves [14]
Taraxerol (**57**)	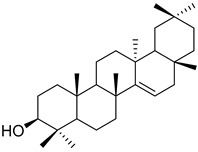	Methanol extract of *Morella adenophora* (Hance) J. Herb. roots [19] Chloroform and methanol extract of *Morella arborea* (Hutch.) Cheek twigs [20] Hexane extract of *Morella cerifera* (L.) Small twigs [23] Benzene extract of *Myrica gale* L. (*Myrica gale* var *tormentosa* L.) stems [82] Benzene extract of *Morella cerifera* (L.) Small (*Myrica cerifera*) root bark [42] Extract of the bark of *Myrica esculenta* Buch.-Ham. ex D. Don [83]
Taraxerone (**58**)	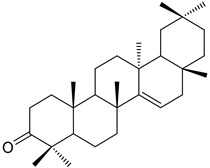	Chloroform and methanol extract of *Morella arborea* (Hutch.) Cheek twigs [20] Benzene extract of *Morella cerifera* (L.) Small (*Myrica cerifera*) root bark [42]
Myricadiol (**59**)	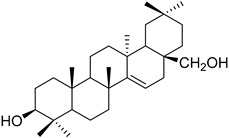	Chloroform and methanol extract of *Morella arborea* (Hutch.) Cheek twigs [20] Benzene extract of *Myrica gale* L. (*Myrica gale* var *tormentosa* L.) stems [82] Hexane extract of *Morella cerifera* (L.) Small twigs [23]
Alphitolic acid (**60**)	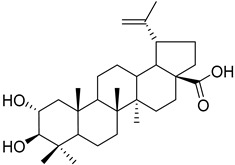	Chloroform and methanol extract of *Morella arborea* (Hutch.) Cheek twigs [20]
Maslinic acid (**61**)	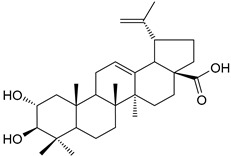	Chloroform and methanol extract of *Morella arborea* (Hutch.) Cheek twigs [20]
Myriceric acid C (**62**)	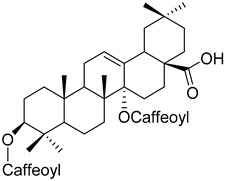	Methanol extract of *Morella cerifera* (L.) Small (*Myrica cerifera*) twigs [60]
Myriceric acid B (**63**)	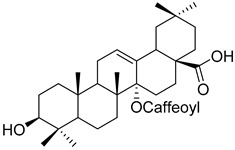	Methanol extract of *Morella cerifera* (L.) Small (*Myrica cerifera*) twigs [60]
Myriceric acid D (**64**)	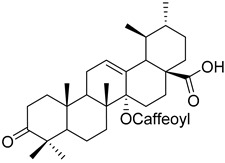	Methanol extract of *Morella cerifera* (L.) Small (*Myrica cerifera*) twigs [60]
Serratenedione (**65**)	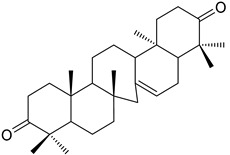	Benzene extract of *Myrica gale* L. (*Myrica gale* var *tormentosa* L.) stems [82]
Serratenediol (**66**)	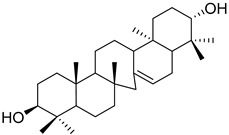	Benzene extract of *Myrica gale* L. (*Myrica gale* var *tormentosa* L.) stems [82]
Myricolal (**67**)	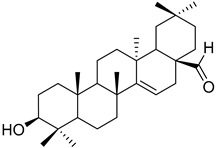	Benzene extract of *Myrica gale* L. (*Myrica gale* var *tormentosa* L.) stems [82]
Arjunglucoside (**68**)	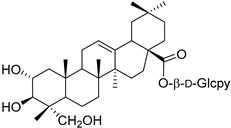	Methanol extract of *Myrica esculenta* Buch.-Ham. ex D. Don leaves [14]
3-*epi*-Ursolic acid (**69**) **	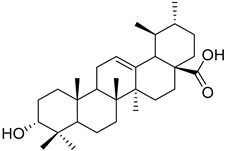	Methanol extract of *Myrica esculenta* Buch.-Ham. ex D. Don leaves [14]
3-*O*-(*E*)-Caffeoyl-ursolic acid (**70**) **	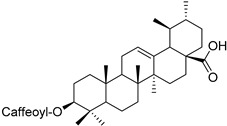	Methanol extract of *Myrica esculenta* Buch.-Ham. ex D. Don leaves [14]

* The stereochemistry was defined by Jahanban-Esfahlan et al. [84]; ** The name was corrected based in the structure presented in the original paper since ursonic acid has a ketone group at C-3; L-Araf = L-arabinofuranosyl; D-Glcpy = D-glucopyranoside; Xyl = D-xylopyranosyl; Galloyl = 3,4,5-trihydroxybenzoyl; Api = −β-D-Apiofuranosyl; Caffeoyl = 3-hydroxycoumaroyl.

## Abbreviations

6-OHDA ACE-1	6-hydroxydopamine Angiotensin-converting-enzyme 1
AGE	Advanced glycation end products
AICAR	5-Aminoimidazole-4-carboxamide ribonucleotide
Akt	Protein kinase B
ALT	Alanine transaminase
AMPK aP2	Adenosine monophosphate-activated protein kinase Adipocyte protein 2
AST	Aspartate transaminase
ATP	Adenosine tri-phosphate
α-SMA	alpha smooth muscle actin
Bax	Bcl-2-associated X
Bcl-2	B-cell lymphoma 2
C/EBPα	CCAAT/enhancer-binding-protein-α
COX-2	Cycloxygenase-2
CYP2E1	Cytochrome P450 2E1
DNP	2,4-dinitrophenol
EC_50_	Half maximal effective concentration
ERK	Extracellular-signal-regulated kinase
FoxOs	Forkhead box O3
γ-GCS	Gamma-glutamylcysteine synthetase
GSK-3β	Glycogen synthase kinase 3 beta
HDL	High-density lipoprotein
HFD	High-fat diet
HIF-1α	Hypoxia-inducible factor 1-alpha
HMGB1	High mobility group box 1 protein
HO-1	Heme oxygenase 1
HUVECs	Human umbilical vein endothelial cells
IC_50_	Half maximal inhibitory concentration
IL-1β	Interleukin-1β
IL-6	Interleukin-6
iNOS	Nitric oxide synthase
IRS-1	Insulin receptor substrate 1
IKK-β	I-kappa-B-kinase beta
JNK	c-Jun NH2-terminal cinase
LDH	Lactate Dehydrogenase
LDL	Low-density lipoprotein
LKB1	Tumor suppressor serine/threonine-protein kinase
LPS	Lipopolysaccharide
MAPK	Mitogen-activated protein kinase
MCP-1	Monocyte chemoattractant protein-1
MIC	Minimum inhibitory concentration
mTOR	mammalian target of rapamycin
MuRF1	Muscle RING-finger protein-1
MyD88	Myeloid differentiation primary response 88
NA	Neuraminidase
NF-_K_B	Nuclear factor kappa B
NQO-1	NAD(P)H Quinone Dehydrogenase 1
Nrf2	Nuclear factor erythroid 2-related factor 2
Ox-LDL	Oxidized low-density lipoprotein
ROS	Reactive oxygen species
PGC-1α	Peroxisome proliferator-activated receptor-gamma coactivator
PI3-K	Phosphoinositide 3-kinases
PPARγ	Peroxisome proliferator-activated receptor gamma
PSD-45	Postsynaptic density protein-45
SIRT1	Sirtuin 1
SREB-1	Sterol regulatory element-binding transcription factor 1
STAT3	Signal transducer and activator of transcription 3
TGF-β1	Transforming growth factor beta 1
TH	Tyrosine hydroxylase
TG	Triglycerides
TLR4	Toll-like receptor 4
TNF-α	Tumor necrosis factor α
VEGF	Vascular endothelial growth factor
